# Long-term treatment outcome of Castleman’s disease: A real-world experience

**DOI:** 10.3389/fonc.2022.974770

**Published:** 2022-08-05

**Authors:** Gi-June Min, Young-Woo Jeon, Tong Yoon Kim, Dae Hun Kwag, Jong Hyuk Lee, Joon Yeop Lee, Sung-Soo Park, Silvia Park, Jae-Ho Yoon, Sung-Eun Lee, Byung-Sik Cho, Ki-Seong Eom, Yoo-Jin Kim, Seok Lee, Hee-Je Kim, Chang-Ki Min, Jong Wook Lee, Seok-Goo Cho

**Affiliations:** ^1^ Department of Hematology, Seoul St. Mary’s Hematology Hospital, College of Medicine, The Catholic University of Korea, Seoul, South Korea; ^2^ Department of Hematology, Yeouido St. Mary’s Hematology Hospital, College of Medicine, The Catholic University of Korea, Seoul, South Korea

**Keywords:** Castleman disease, interleukin-6, age, splenomegaly, siltuximab, steroids

## Abstract

**Background:**

Castleman disease (CD), classified as unicentric CD (UCD) or multicentric CD (MCD), is a rare non-neoplastic lymphoproliferative disorder of unknown origin. Owing to its rarity, the clinical characteristics, therapeutic modalities, treatment outcomes, and prognostic factors related to UCD or MCD are not well defined.

**Method:**

We retrospectively analyzed 88 patients with CD, including those with hyaline-vascular, plasma-cell, mixed type, hypervascular, and plasmablastic subtypes, for presenting symptoms, physical, laboratory, and radiologic findings, and treatment response in the Korean population.

**Results:**

The median patient age was 44 years (range: 18–84 years) with slight predominance of women (53.4%). UCD and MCD accounted for 38.6% (n=34) and 61.4% (n=54) of cases, respectively. Histopathologically, UCD patients were classified as 88.2% (n=30) hyaline-vascular and 11.8% (n=4) plasma cell types, whereas MCD patients were classified as 27.8% (n=15) hypervascular, 61.1% (n=33) plasma cell, 7.4% (n=4) mixed, and 3.7% (n=2) plasmablastic types. Twelve (13.6%) patients exhibited a poor performance status with an Eastern Cooperative Oncology Group score of 2. The most common presenting symptom was sustained fever, followed by fatigue, anorexia, peripheral edema, and weight loss. Furthermore, splenomegaly, pleural effusion, and ascites were observed to be associated with CD. Surgical resection and siltuximab were the preferred treatment modalities for UCD and MCD, respectively, with favorable symptomatic, laboratory, and radiologic outcomes and safety profiles. The overall survival was 90.2%, with no significant difference between the UCD and MCD groups (p=0.073), but progression-free survival was significantly poorer in the MCD group (p=0.001). Age ≥60 years and splenomegaly significantly affected the overall and progression-free survival rates.

**Conclusion:**

Patients with UCD had favorable outcomes with surgical resection of a solitary mass, whereas in patients with MCD, old age and splenomegaly were identified as independent prognostic factors. Further well-designed prospective studies under advancing knowledge of the pathophysiology of MCD are warranted to establish suitable guidelines for the discontinuation or prolonging infusion intervals of siltuximab and treatment modalities for HHV-8 positive MCD patients or patients with siltuximab failure.

## Introduction

Castleman disease (CD) is a rare non-neoplastic lymphoproliferative disorder of unknown origin that was first described in 1956 (1). The clinical manifestations and management strategies for CD are distinct and vary with the clinical and pathological subtypes. CD is histopathologically classified into hyaline-vascular, plasma-cell, mixed type, hypervascular, and plasmablastic phenotypes (2). Based on the lesions involved, CD presents as unicentric CD (UCD; localized form) or multicentric CD (MCD; systemic form). UCD is typically indolent without systemic symptoms, and complete surgical resection of the localized mass is considered the gold standard treatment with a long-lasting response (3). Unlike UCD, MCD presents with multiple peripheral lymphadenopathies and systemic symptoms such as fever, night sweats, weight loss, or fatigue, and its manifestation essentially results from proinflammatory hypercytokinemia of interleukin-6 (IL-6) (4). Given the rarity of CD, most studies are retrospective or case reports, and the clinical characteristics, therapeutic modalities, treatment outcomes, and prognostic factors related to UCD or MCD are not well defined until now. Evidence suggests that there are distinct regional differences in the etiology of CD in (5, 6), and its clinicopathologic characteristics have been investigated in several countries, including the US (5), Japan (6, 7), and France (8). However, no study has investigated the clinical characteristics and prognosis of CD in Korea. Furthermore, most retrospective data were collected before siltuximab became the standard treatment modality for MCD. Although siltuximab treatment response is efficient in either first-line or second-line therapy, there are currently no prospective studies on the extension or termination of siltuximab treatment after complete remission of MCD; there are currently no effective salvage treatment modalities after siltuximab failure. Therefore, it is essential to develop additional clinically appropriate treatment guidelines through prospective studies. The long-term clinical outcomes and adverse event profile of siltuximab are also of interest. This study aimed to elucidate the clinical characteristics, long-term survival outcomes, and prognoses of various CD subtypes.

## Materials and methods

### Patient enrollment and diagnosis

This retrospective study enrolled 88 patients diagnosed with CD between January 2006 and December 2020 at St. Mary’s Hematology Hospital in Seoul, South Korea. Expert pathologists confirmed and cross-checked all histopathological features of CD diagnoses, including abnormal, regressed, or hyperplastic germinal centers, follicular dendritic cell predominance, hypervascularization, mantle zone expansion, and interfollicular plasmacytosis.

Initial physical examination and presenting symptoms were thoroughly reviewed, especially the IL-6-related inflammatory responses caused by CD, such as sustained fever, fatigue, anorexia, peripheral edema, or weight loss. These symptoms were carefully assessed based on the National Cancer Institute Common Terminology Criteria for Adverse Events (NCI-CTC-AE) version 4.0. Laboratory examinations included complete blood count with differential count, C-reactive protein (CRP), erythrocyte sedimentation rate (ESR), creatinine, total protein, albumin, and β2-microglobulin levels at the time of CD diagnosis. Epstein-Barr virus (EBV), cytomegalovirus (CMV), human immunodeficiency virus (HIV), and human herpesvirus type 8 (HHV-8) infections were assessed using real-time reverse transcription-polymerase chain reaction (RT-PCR) in plasma or paraffin-embedded tissue samples. Patients with any evidence of significant infection, including hepatitis B or C, concurrent lymphoma, and multiple myeloma were excluded.

All enrolled patients underwent radiological imaging, including computed tomography (CT) of the neck, chest, abdomen, and pelvis, to identify and measure lymphadenopathies or organs and other disease-related features, including splenomegaly, ascites, or pleural effusion. The patients also underwent systemic 18-fluoro-2-deoxy-D-glucose (FDG) positron emission tomography (PET)-CT torso scan at the time of diagnosis for the initial staging workup and to rule out other combined malignancies. Based on radiological findings, UCD was defined as solitary lymphadenopathy, characterized by the distribution of affected lymph nodes at a single site, or a single extranodal lesion that was surgically resectable, whereas MCD was defined as the involvement of two or more lymph nodes or regions.

MCD can be further classified as POEMS (polyneuropathy, endocrinopathy, monoclonal gammopathy, and skin change)-associated, HHV-8-associated, and idiopathic MCD (iMCD) (9). Patients with HIV-negative and HHV-8-negative MCD of unknown etiology and pathophysiology were considered to have iMCD. Among iMCD cases, severe iMCD diagnosis must satisfy at least two of the following five criteria: (A) Eastern Cooperative Oncology Group (ECOG) performance status score ≥2; (B) stage IV renal dysfunction (estimated glomerular filtration rate [eGFR] <30 or serum creatinine >3.0); (C) anasarca and/or ascites and/or pleural/pericardial effusion; (D) hemoglobin level ≤8.0g/dL; and (E) pulmonary involvement and/or interstitial pneumonitis with dyspnea (9). Patients were classified as having non-severe iMCD if none of the five criteria were met.

### Treatment strategy and clinical outcomes

All patients received the best supportive care, including constitutional symptom management using antipyretics, antipruritics, antihistamines, and pain medications. If an active infection was detected during the diagnosis of CD, antibiotics were aggressively administered. The available CD treatment modalities were observation, surgical resection, radiotherapy, steroid pulse therapy, CHOP chemotherapy regimen consisting of cyclophosphamide (750 mg/m^2^), vincristine (1.4 mg/m^2^), and rituximab (375 mg/m^2^) on day 1 and prednisolone (60 mg/m^2^) on days 1–5, or intravenous siltuximab 11mg/kg/day infusion every 21 days. Patients receiving steroid pulse therapy were administered methylprednisolone (1.0 mg/kg/day) by intravenous or oral route for seven days, with a weekly dose reduction of 20%. During steroid therapy, clinical symptoms and laboratory values were thoroughly evaluated weekly. In the case of radiotherapy, the total treatment dose was 27–30 Gy with a daily fraction size of 1.8–2.0 Gy. The radiation dose was determined based on the location and extent of CD lesions. All patients who underwent CHOP chemotherapy were subjected to six cycles at three-week intervals, and an imaging workup was performed after the end of the third and sixth cycles. If the patient achieved complete remission (CR) after the sixth CHOP therapy cycle, follow-up evaluations were performed at six-month intervals for five years. Siltuximab-treated patients underwent a three-week interval of continuous infusion until treatment failure, which was defined as newly appearing disease-related NCI-CTC-AE grade ≥3 symptoms, persistence of grade ≥2 disease-related symptoms for more than three weeks, ECOG score elevation by more than one point, persistence for at least three weeks, and radiological progression.

Treatment response was assessed according to the Castleman Disease Collaborative Network (CDCN) consensus guidelines (9). There are three composite endpoints: (A) four laboratory parameters of inflammatory response and organ function, including hemoglobin, CRP, albumin, and eGFR, which can be broken down to blood urea nitrogen and serum creatinine levels; (B) four crucial clinical symptoms of fatigue, anorexia, fever, and weight change; and (C) lymph node size. Overall CR requires normalization of clinical symptoms, biochemical markers, and CR in lymph node response that was assessed using the modified Cheson criteria (10, 11). Partial remission (PR) required a decrease of more than one NCI-CTC-AE grade point in clinical symptoms, more than 50% improvement in all biochemical markers (but not to baseline), and PR in lymph node response. Stable disease (SD) was defined as no CR, PR, or progressive disease (PD) criteria. PD required >50% worsening in any biochemical markers, any worsened symptoms on two subsequent assessments, and >25% increase in the affected lymph node response. The overall response rate (ORR) was defined as the overall number of patients who achieved CR or PR.

### Statistical analysis

All statistical analyses were performed using R software version 3.4.1 (R Foundation for Statistical Computing, 2017). Statistical significance was set at *p*<0.05 and all *p* values were two-sided. Chi-square or Fisher’s exact tests were used to compare categorical variables. Student’s *t*-test or Mann–Whitney U test was used to compare the continuous variables. Overall survival (OS) and progression-free survival (PFS) were defined as the length of time from CD diagnosis to the last follow-up before death, regardless of the disease status, stable disease, or status quo in the treatment modality. The Kaplan–Meier method was used to estimate OS and PFS, and log-rank analysis was used to compare the survival distributions. The multivariate model was derived using step-wise selection among candidate variables from univariate analysis; the Wald test was used for the overall *p*-value for factors with >2 levels and *p*-value <0.05 to warrant inclusion in the model.

### Ethics approval and consent to participate

After receiving approval from the Institutional Review Board (IRB) of Seoul St. Mary’s Hospital (KC22RASI0400), all retrospective analyses were performed according to the Institutional Review Board guidelines and the tenets of the Declaration of Helsinki. The need for informed consent was waived owing to the retrospective nature of the study, with approval from the IRB.

## Results

### Clinical characteristics

The baseline characteristics of patients with CD are presented in **Table 1**. The median patient age was 44 years (range, 18–84 years), with a relative predominance of female patients (n=47, 53.4%). The number of UCD and MCD patients were 34 (38.6%) and 54 (61.4%), respectively. Histopathologically, UCD patients were classified as 88.2% (n=30) hyaline-vascular and 11.8% (n=4) plasma cell types, whereas MCD patients were classified as 27.8% (n=15) hypervascular, 61.1% (n=33) plasma cell, 7.4% (n=4) mixed, and 3.7% (n=2) plasmablastic types. In the MCD group, 50 patients were identified as having iMCD and 32 of them had severe iMCD. Two patients were identified as having POEMS-associated MCD, and the other two were identified as HHV-8 positive. None of the HIV-positive patients with MCD were enrolled in this study. A detailed classification of CD in the enrolled patients is shown in **Supplementary Figure 1**. Twelve (13.6%) patients had a poor performance status (ECOG score 2), mostly owing to CD-related inflammatory signs or symptoms. The most common presenting symptom of CD was sustained fever (n=34, 38.6%), followed by fatigue (n=22, 25.0%), anorexia (n=15, 17.0%), peripheral edema (n=14, 15.9%), and weight loss (n=13, 14.8%). Splenomegaly (n=21, 23.9%), pleural effusion (n=13, 14.8%), ascites (n=11, 12.5%), and pulmonary involvement (n=5, 5.7%) were associated with CD.

**Table 1:** Baseline characteristics of patients with Castleman disease (n=88).

Characteristics	Number of patients (%)
**Age, median (range)**	44 (18–84) years
≥60 years	13 (14.8%)
**Sex**	
Male	41 (46.6%)
Female	47 (53.4%)
**ECOG**	
0	27 (30.7%)
1	49 (55.7%)
2	12 (13.6%)
**Disease-related symptoms**	
Sustained fever	34 (38.6%)
Fatigue	22 (25.0%)
Anorexia	15 (17.0%)
Peripheral edema	14 (15.9%)
Weight loss	13 (14.8%)
**Disease-related signs**	
Splenomegaly	21 (23.9%)
Pleural effusions	13 (14.8%)
Ascites	11 (12.5%)
Pulmonary involvement (GGOs)	5 (5.7%)
**Disease histopathology**	
UCD	N=34
Hyaline vascular type	30 (88.2%)
Plasma cell type	4 (11.8%)
MCD	N=54
Hypervascular type	15 (27.8%)
Plasma cell type	33 (61.1%)
Mixed type	4 (7.4%)
Plasmablastic type	2 (3.7%)
**Classification of Castleman Disease**	
UCD	34 (38.6%)
MCD †	54 (61.4%)
**UCD Localization**	
Abdominal	13 (38.2%)
Cervical	6 (17.7%)
Axillar	6 (17.7%)
Mediastinal	5 (14.6%)
Inguinal	2 (5.9%)
Extranodal	2 (5.9%)
**Laboratory findings, median (range)**	
Hemoglobin (g/dL)	12.9 (4.0–16.0)
C-reactive protein (mg/L)	0.20 (0.01–24.65)
Erythrocyte sedimentation rate (mm/h)	12.0 (2.0–120.0)
Serum creatinine (mg/dL)	0.76 (0.25–2.14)
Total protein (g/dL)	7.2 (5.3–11.9)
Albumin (g/dL)	4.1 (1.9–5.0)
Lactate dehydrogenase (g/dL)	343.5 (117.0–2095.0)
Ferritin (ng/mL)	136.6 (8.7–2899.0)

ECOG, European Cooperative Oncology Group; GGOs, ground-grass opacities; MCD, multicentric Castleman disease; UCD, unicentric Castleman disease.

†The detailed subtype of MCD is presented in **Supplementary Figure 1**.

A comparison of the clinical characteristics between the 34 and 54 patients with UCD (median age of 41.5, range 18–76) and MCD (median age of 45.5, range 21–84), respectively, is shown in **Table 2**. Most patients with MCD were male (57.4% vs. 29.4%, *p*=0.010) and had a poor ECOG performance status score of 2 (18.5% vs. 5.9%, *p*<0.001), splenomegaly (35.2% vs. 5.9%, *p*=0.002), and pleural effusion (24.1% vs. 0%, *p*=0.001). All patients with POEMS syndrome (n=2) and HHV-8-positive patients (n=2) were included in the MCD group. Except for the lactate dehydrogenase (LDH) level, hemoglobin, CRP, ESR, serum creatinine, total protein, albumin, and ferritin levels were worse in the MCD group.

**Table 2:** Comparison of clinical characteristics and survival outcomes between patients with UCD and MCD.

Characteristics	UCD (n=34) N (%)	MCD (n=54) N (%)	*p* value
**Age, ≥60 years**	2 (5.9%)	11 (20.4%)	0.062
**Sex, male**	10 (29.4%)	31 (57.4%)	0.010
**ECOG 2**	2 (5.9%)	10 (18.5%)	<0.001
**Disease-related signs**			
Splenomegaly	2 (5.9%)	19 (35.2%)	0.002
Pleural effusions	0 (0%)	13 (24.1%)	0.001
Ascites	3 (8.8%)	8 (14.8%)	0.408
Pulmonary involvement (GGOs)	0 (0%)	5 (9.3%)	0.068
**Disease-related symptoms**			
Sustained fever	2 (5.9%)	32 (59.3%)	<0.001
Fatigue	2 (5.9%)	20 (37.0%)	0.001
Anorexia	0 (0%)	15 (27.8%)	0.001
Peripheral edema	1 (2.9%)	13 (24.1%)	0.008
Weight loss	2 (5.9%)	11 (20.4%)	0.062
**HHV-8 positive**	0 (0%)	2 (3.7%)	0.520
**POEMS syndrome** †	0 (0%)	2 (3.7%)	0.520
**Laboratory findings, mean (± S.E.)**			
Hemoglobin (g/dL)	13.6 ± 0.3	11.4 ± 0.4	<0.001
C-reactive protein (mg/L)	0.21 ± 0.103	4.04 ± 0.78	<0.001
Erythrocyte sedimentation rate (mm/h)	6.2 ± 1.4	39.9 ± 4.9	<0.001
Serum creatinine (mg/dL)	0.71 ± 0.03	0.85 ± 0.05	0.028
Total protein (g/dL)	7.2 ± 0.1	7.7 ± 0.2	0.041
Albumin (g/dL)	4.5 ± 0.1	3.6 ± 0.1	<0.001
Lactate dehydrogenase (g/dL)	338.6 ± 16.3	416.2 ± 42.3	0.161
Ferritin (ng/mL)	127.3 ± 22.5	335.1 ± 76.9	0.032
**Overall survival**	100%	85.8% (69.2–93.9)	0.073
**Progression free survival**	100%	45.3% (21.9–66.2)	0.001

ECOG, European Cooperative Oncology Group; HHV-8, Human herpesvirus 8; MCD, multicentric Castleman disease; UCD, unicentric Castleman disease.

† POEMS syndrome consists of polyneuropathy, organomegaly, endocrinopathy, monoclonal gammopathy, and skin changes.

### Treatment modalities and outcomes in patients with CD

**Figure 1:**
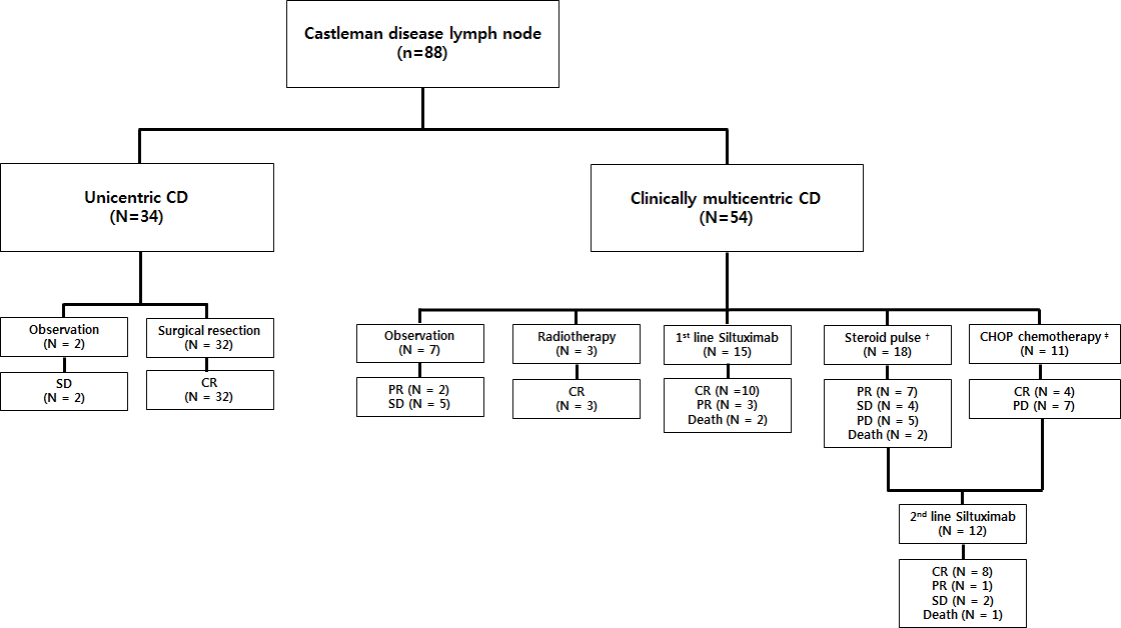
Treatment outcomes of patients with CD (N=88). Two patients with UCD and 7 with MCD were observed without any treatment after diagnosis. Among these nine patients, seven had SD. Thirty-two patients with UCD underwent surgery and achieved CR (ORR, 100%). Three patients with localized MCD received radiotherapy and achieved CR (ORR, 100%). Forty-four patients underwent systemic therapy: 18 received steroid pulse therapy (ORR 38.9%), 11 received CHOP chemotherapy (ORR 36.4%), and 15 received frontline siltuximab infusion (ORR 86.7%). Five patients from the steroid-treated group and seven from the CHOP chemotherapy group, who exhibited PD, received salvage siltuximab infusion (ORR 75.0%). All deceased patients were due to MCD disease progression. † Steroid pulse therapy represents receiving methylprednisolone ≥ 1.0 mg/kg/day. ‡ CHOP chemotherapy consisted of cyclophosphamide (750 mg/m^2^), vincristine (1.4 mg/m^2^), rituximab (375 mg/m^2^) on day 1, and prednisolone (60 mg/m^2^) on days 1–5 every 21 days, or intravenous siltuximab infusion. CD, Castleman disease; SD, stable disease; CR, complete remission; PR, partial remission; PD, progressive disease; UCD, unicentric Castleman disease; MCD, multicentric Castleman disease; ORR, overall response rate.

The treatment modalities of patients with CD in this study and their clinical outcomes are presented in **Figure 1**. Among the 34 patients with UCD, 32 underwent initial surgical resection of solitary lymphadenopathy as treatment, and all achieved CR. The remaining two patients were diagnosed by core-needle biopsy and underwent long-term observation due to refusal of surgical resection and lack of systemic symptoms and laboratory abnormalities, despite the presence of a stable nodal mass on CT. They were regularly followed up with laboratory and imaging tests every six months, and SD was maintained. Furthermore, seven asymptomatic patients without complications in the MCD group underwent observation using the “watch and wait” strategy. Five patients showed SD, and the rest showed PR of lymphadenopathy without systemic inflammatory symptoms. Three patients initially presented with multiple lymphadenopathies that were limited to the cervical area, underwent local radiotherapy, and achieved CR. Total 44 patients underwent systemic therapy: 18 with steroid pulse therapy, 11 with CHOP chemotherapy, and 15 with three-week interval first-line siltuximab infusion. Among the 18 patients who received steroid pulse therapy, seven, four, and seven showed PR, SD, and PD, respectively. Moreover, 5 of the 7 patients with PD received second-line siltuximab therapy, but the remaining 2 died because of combined septic shock. Among the 11 patients treated with a CHOP regimen, 4 achieved CR, and 7 received second-line siltuximab therapy owing to PD.

### Treatment outcomes of patients with MCD treated with siltuximab

**Figure 2:**
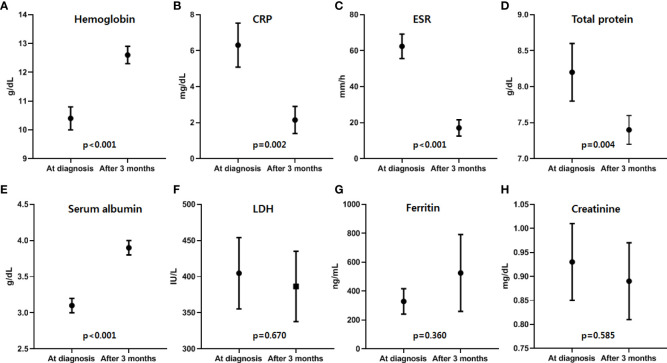
Laboratory responses after the first three months of siltuximab treatment. Among the responders, improvements in laboratory parameters, including four laboratory parameters of inflammatory response suggested by CDCN guideline 2018, of MCD were observed after a median of 3.2 months (range, 2.8-8.2), and **(A)** hemoglobin, **(B)** CRP, **(C)** ESR, **(D)** total protein, and **(E)** albumin levels showed significant improvement after siltuximab treatment. However, there was no significant difference in **(F)** LDH, **(G)** ferritin, and **(H)** creatinine levels (Black dots and lines represent the median values and ranges of each parameter). Hb, hemoglobin; CRP, C-reactive protein; ESR, erythrocyte sedimentation rate; LDH, lactate dehydrogenase; CDCN, the Castleman Disease Collaborative Network; MCD, multicentric Castleman disease.

**Figure 3:**
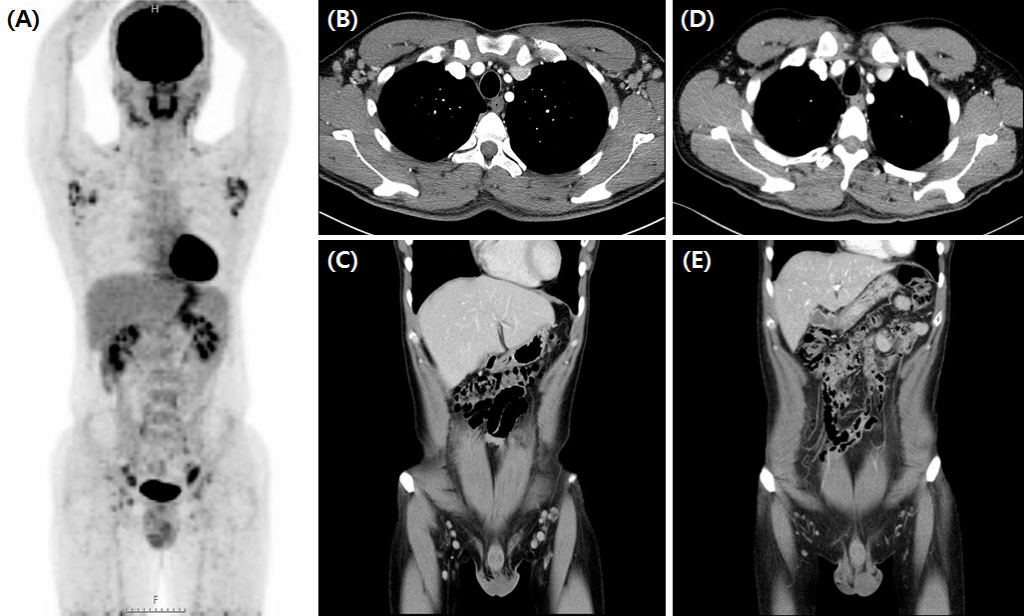
Significant regression of enlarged lymph nodes after third siltuximab infusion. Initial FDG-PET CT scan of iMCD plasma cell type patient **(A)** showed MCD involving bilateral cervical, supraclavicular, axillae, common and external iliac, and inguinal lymph nodes. CT scan findings before **(B, C)** and after **(D, E)** siltuximab infusion showed markedly regressed MCD-involved lymph nodes in the bilateral inguinal and axillar areas. FDG-PET CT, 18-fluoro-2-deoxy-D-glucose positron emission tomography-computed tomography; iMCD, idiopathic multicentric Castleman disease.

Siltuximab treatment, an anti-IL-6 therapy used to treat MCD in patients negative for HIV and HHV-8 and proven to improve clinical outcomes, was used in patients with MCD exhibiting systemic symptoms and laboratory abnormalities. Among them, 55.6% (n=15) were treated with siltuximab as first-line therapy and 44.4% (n=12) were treated as second-line therapy after previous CHOP chemotherapy (n=7) or steroid pulse therapy (n=5). CR was observed in 22 (81.5%) patients, and a durable symptomatic response was achieved at a median of 23.5 days (range, 15–82 days) after the initiation of siltuximab treatment. On laboratory evaluation after three months of treatment, the median values of hemoglobin (range, 10.4–12.6 g/dL; *p*<0.001), CRP (range, 6.31–2.15 mg/dL, *p*=0.002), ESR (range, 62.4–17.1 mm/h; *p*<0.001), serum total protein (range, 8.2–7.4 g/dL; *p*=0.004), and serum albumin (range, 3.1–3.9 g/dL; *p*<0.01) were significantly improved (**Figure 2**). One patient demonstrated a dramatic regression of multifocal lymphadenopathies in imaging studies after the third siltuximab infusion (**Figure 3**). At a median of 12.9 months (range, 5.5–93.0 months) after the initiation of siltuximab, 66.7% (n=18) and 14.8% (n=4) of patients achieved CR and PR, respectively. Of the siltuximab responders who achieved CR or PR (81.5%), all achieved CR in terms of clinical symptoms and laboratory parameters before the radiologic response. However, five patients (18.5%) did not show a proper response during siltuximab treatment, and three had systemic symptoms, abnormalities in laboratory markers, and unresponsive multiple lymphadenopathies despite siltuximab infusion. The remaining two patients discontinued treatment with siltuximab with SD. One patient refused treatment due to poor general condition after the second cycle of siltuximab, and the other patient changed treatment to CHOP chemotherapy for faster resolution of the lymphoproliferative mass compressing the right ureter despite significant symptomatic improvement after four cycles of siltuximab. On average, improvements in clinical symptoms, laboratory parameters, and radiologic parameters of MCD among responders were observed after one, three, and 18 cycles of siltuximab treatment, respectively. The clinical responses of siltuximab-treated patients with MCD (n=27) are presented in **Table 3**.

**Table 3:** Clinical responses of siltuximab-treated patients with MCD (n=27).

Clinical response	All patients
**Duration of treatment, median (range)**	39 cycles (2–173)
**Symptomatic response, n (%)**	22 (81.5%)
**Time to durable symptomatic response, days (range)**	23.5 days (15–82)
**Laboratory response at 3 months, median (range)**	
Hemoglobin (g/dL)	12.4 (9.4–16.0)
C-reactive protein (mg/L)	0.15 (0.01–14.57)
Erythrocyte sedimentation rate (mm/h)	5.0 (2.0–71.0)
Serum creatinine (mg/dL)	0.84 (0.07–2.35)
Total protein (g/dL)	7.4 (5.4–10.1)
Albumin (g/dL)	4.0 (2.5–5.0)
Lactate dehydrogenase (g/dL)	320.0 (151.0–1429.0)
Ferritin (ng/mL)	108.3 (31.2–5054.0)
**Overall response, n (%)** †	
*Responder*	*81.5%*
Complete remission	18 (66.7%)
Partial remission	4 (14.8%)
*Non-responder*	*18.5%*
Stable disease	2 (7.4%)
Disease progression	3 (11.1%)
**Time to response (complete or partial remission), median (range)**	12.9 months (5.5–93.0)
Adverse events, n (%)*	
Upper respiratory infection, Grade I–II ‡	6 (22.3%)
Maculopapular rash, Grade II	4 (14.8%)
Peripheral neuropathy, Grade II	3 (11.2%)
Hepatopathy, Grade III	3 (11.1%)
Nephropathy, Grade III	2 (7.4%)
Neutropenia, Grade II	2 (7.4%)
Diarrhea, Grade II	1 (3.7%)
Weight gain, Grade II	1 (3.7%)
Reactivation of pulmonary tuberculosis	1 (3.7%)

† Imaging responses were evaluated based on the Cheson criteria (selected owing to the lymphoproliferative nature of multicentric Castleman disease) for computed tomography images of the neck, abdomen, and chest.

*Toxicity was evaluated according to the NCI CTC-AE (National Cancer Institute Common Terminology Criteria for Adverse Events) guidelines version 4.0.

‡ Since the first outbreak of coronavirus disease-19 in January 2020 in Korea, two upper respiratory infection patients revealed polymerase chain reaction positive in severe acute respiratory syndrome virus type 2 during siltuximab treatment. However, their symptoms were mild without any severe adverse events.

### Adverse events of siltuximab treatment

Siltuximab demonstrated a favorable safety profile and prolonged treatment was well tolerated. During siltuximab treatment, the most common adverse events were upper respiratory tract infections (n=6, 22.3%) with mild to moderate symptoms, followed by NCI-CTC-AE grade 2 maculopapular rash (n=4, 14.8%), peripheral neuropathy (n=3, 11.2%), neutropenia (n=2, 7.4%), diarrhea (n=1, 3.7%), and weight gain (n=1, 3.7%). Since the first outbreak of coronavirus disease-19 (COVID-19) in January 2020 in Korea, two upper respiratory infection patients revealed polymerase chain reaction positive in severe acute respiratory syndrome virus type 2 (SARS-CoV-2) during siltuximab treatment. However, their symptoms were mild without any severe adverse events. One patient experienced reactivation of pulmonary tuberculosis after the initial presentation with cough and yellow sputum. More severe adverse events of grade 3 hepatopathy or nephropathy were observed in three (11.2%) and two (7.4%) patients, respectively.

### Survival outcomes and prognostic factors

**Figure 4:**
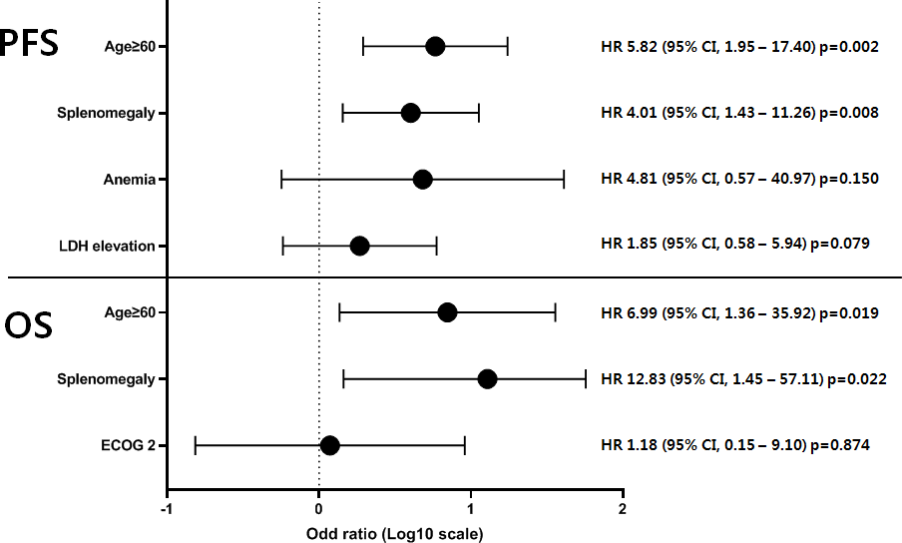
Multivariate analysis of prognostic factors in 54 patients with MCD. The prognostic factors of 54 patients with MCD showing clinical significance in univariate analysis were subjected to multivariate analysis using a Cox regression model. As a result, age ≥60 years and splenomegaly significantly affected the survival outcomes in terms of both OS and PFS. PFS, progression-free survival; OS, overall survival; HR, hazard ratio; CI, confidence interval; MCD, multicentric Castleman disease.

During a median follow-up of 53.5 months (range, 4.0–192.0 months), the OS rate was 90.2% (95% confidence interval [CI], 77.1–96.0). In our cohort, there was no significant difference in OS between the UCD and MCD groups (100% vs. 85.8%, *p*=0.073), but PFS was significantly poor in the MCD group (100% vs. 45.3%, *p*=0.001). Prognostic factors showing clinical significance in the univariate analysis were subjected to multivariate analysis using a Cox regression model (**Figure 4**). In the univariate analysis, old age (≥60 years), poor performance status (ECOG score of 2), and splenomegaly were risk factors related to patients with MCD. Consequently, age ≥60 years (hazard ratio [HR)] 6.99, 95% CI, 1.36–35.92, *p*=0.019) and splenomegaly (HR 12.83, 95% CI, 1.45–57.11, *p*=0.022) significantly affected OS. Furthermore, age ≥60 years (HR 5.82, 95% CI, 1.95–17.40, *p*=0.002) and splenomegaly (HR 4.01, 95% CI, 1.43–11.26, *p*=0.008) were significantly associated with inferior PFS. Multivariate analysis was conducted with the significant univariate factors of age ≥60 years, splenomegaly, anemia, and LDH elevation at the time of CD diagnosis. The results of the univariate analysis are presented in **Supplementary Table 1**.

## Discussion

Due to the rarity of CD, reports on the clinical characteristics and treatment outcomes of UCD are limited to a few case series or retrospective studies with no controls, and no studies have investigated the clinical characteristics and prognosis of CD in the Korean population (8, 12–17). In our study, 34 patients with UCD were compared with 54 patients with MCD. Most cases were asymptomatic with better ECOG performance scores. In total, 32 patients underwent surgical resection of solitary lymphadenopathy (94.1% lymph node and 5.9% extranodal mass) as the gold standard treatment for UCD, and all achieved long-term overall CR. Close observation was considered in two asymptomatic patients who remained in a stable state. Notably, two patients exhibiting the plasma cell type UCD with systemic inflammatory symptoms showed an overall CR after surgical resection. Although plasma cell type UCD behaves like MCD and is symptomatic, complete resection provides confirmative diagnosis, eliminates associated systemic symptoms, and is curative (3, 15, 18). If patients with UCD present with surgically unresectable or refractory lesions after initial surgery, debulking of the primary lesion might be considered to reduce local symptoms, and alternative treatments such as radiotherapy, steroid pulse therapy, chemotherapy with or without rituximab, or tocilizumab can be considered to alleviate systemic symptoms (4, 12, 19, 20). However, there is currently no consensus on the optimal treatment strategy for unresectable UCD.

Compared to UCD, patients with MCD showed male predominance, poor ECOG performance score, and a more frequent plasma cell type histopathologic subtype, which was previously evident. Moreover, patients commonly presented with constitutional symptoms, laboratory abnormalities, splenomegaly, ascites, or pleural effusions. Currently, the first-line treatment for MCD, especially iMCD, is siltuximab, as suggested by the 2018 CDCN treatment guidelines (9). Steroid pulse therapy is a monotherapy that is efficient in controlling constitutional symptoms but is short-termed owing to tapering doses (17, 21). CHOP and other cytotoxic chemotherapies based on lymphoma or multiple myeloma regimens can induce acceptable responses but are likely to cause infectious complications and relapse (13, 14, 16, 22, 23). Additionally, rituximab monotherapy has been proven effective only in HIV-positive patients with MCD (24, 25). Siltuximab was unavailable in Korea as a salvage regimen or first-line treatment for MCD before December 2015 and February 2018, respectively. Therefore, CHOP chemotherapy with or without steroid pulse therapy was usually administered in severe cases during the said time period. Regarding clinical outcomes, MCD showed a significantly inferior OS than UCD in several previous studies (13, 14, 16, 22, 23). These studies were retrospective, and most of the analyzed data were collected before siltuximab became a standard treatment modality for MCD and with limited use of anti-IL-6 therapy. However, siltuximab treatment has been proven to improve the survival outcomes of MCD, which might have resulted in no significant difference in OS between UCD and MCD (26, 27).

In our study, the ORR values for steroid pulse therapy and CHOP chemotherapy were 38.9% and 36.4%, respectively. However, the siltuximab group (either first-line or salvage) showed an ORR of 81.5%, and 18.5% were non-responders during the siltuximab treatment with a median of 39 cycles. Treatment response was equally efficient in either first-line or second-line therapy. With siltuximab treatment, most of the patients’ MCD-related constitutional symptoms were resolved, and they were able to independently perform their activities of daily living, which improved their quality of life, even in patients who have achieved PR. MCD patients with lung involvement who experienced severe dyspnea on exertion had recovery of their physical function and were able to engage in vocational activities after siltuximab treatment. Prolonged siltuximab therapy was well tolerated and efficient, suggesting that no neutralizing anti-drug antibodies against siltuximab were formed during the treatment period (28). One MCD patient receiving siltuximab for 118 cycles with 3-week intervals had to discontinue the treatment because she was about to give birth; this led to the recurrence of her constitutional symptoms, but re-administration of siltuximab immediately resolved the MCD-related symptoms.

Siltuximab also demonstrated a favorable safety profile during prolonged treatment; severe adverse events of grade 3 hepatopathy or nephropathy developed in three (11.2%) and two (7.4%) patients, respectively. However, considering the mechanism of action of siltuximab, which binds to IL-6 as an anti-IL-6 chimeric monoclonal antibody and interferes with IL-6 mediated growth of B-lymphocytes, plasma cells, and secreting vascular endothelial growth factor, siltuximab may lower a patient’s resistance to infections (28). One patient was initially diagnosed with pneumonia, which later turned out to be a pulmonary tuberculosis reactivation. Given the high prevalence rate of pulmonary tuberculosis in Korea, efficient monitoring and routine prophylaxis protocol are crucial. Similarly, chronic hepatitis B reactivation, due to siltuximab usage, should also be considered in chronic hepatitis B high prevalent nations such as Korea; we routinely use a prophylaxis dose of oral Entecavir 0.5 mg daily in patients with positive core antibody IgG (HBc-Ab IgG positive). Moreover, siltuximab could inhibit the acute inflammation symptomatology by reducing fever and the level of acute-phase reactants, such as C-reactive protein. Therefore, patients should be monitored for the presence of severe infection by all means. We recommend a thorough work-up for latent infection before commencing siltuximab treatment along with administration of proper prophylactic agents during treatment. Preemptive antibiotic treatment for the possible development of inflammation is also strongly recommended.

Although siltuximab has been proven to be safe and effective for MCD patients, growing evidence demonstrates that siltuximab is also effective for improving the condition of COVID-19 patients in the inflammatory phase. The excessive cytokine release, especially pro-inflammatory cytokine IL-6, is the leading cause of SARS-CoV-2-induced deaths (28–31). In this cohort, two patients with mild upper respiratory symptom were SARS-CoV-2 positive, as detected by polymerase chain reaction testing, during the pandemic, but they did not develop severe respiratory complications during the siltuximab infusion cycles. This supports the beneficial effects of siltuximab in decreasing local and systemic inflammation, which were manifested as enhanced survival and pulmonary function in patients with severe COVID-19 (28–31).

Currently, there are no widely accepted prognostic factors for MCD. A recent relatively large retrospective cohort study reported that age >40 years, splenomegaly, and hypoalbuminemia were independent prognostic factors for MCD (22). Chronological age is a well-known prognostic factor for various lymphoproliferative diseases, including lymphoma and CD; our study showed that age >60 years was significantly associated with poor prognosis in both OS and PFS. Splenomegaly is another previously reported prognostic factor for CD and is commonly present in patients suffering from constitutional symptoms or laboratory abnormalities. Our study also showed a poor prognosis in 35.2% of patients with MCD presenting with splenomegaly. However, laboratory findings, including hypoalbuminemia, decreased renal function, and poor performance status reflected by the ECOG score were not related to MCD prognosis. Paraneoplastic pemphigus is a rare CD-associated cutaneous disorder that is more common in plasma cell type MCD and was recently identified as a prognostic factor for MCD (14, 32, 33). Unfortunately, patients with CD and paraneoplastic pemphigus were not enrolled in this study.

A new disease concept initially recognized as a unique variant of iMCD, known as TAFRO syndrome, was first described by Takai *et al.*, and is characterized by thrombocytopenia, fever, renal dysfunction, organomegaly, and anasarca including generalized edema, ascites, and/or pleural effusions (34). Due to similarity in histopathologic features and elevated expression of IL-6 in patients with MCD and TAFRO syndrome, TAFRO syndrome was described as a related disorder to MCD (35, 36). According to recently published CDCN evidence-based consensus diagnostic criteria, the hyaline-vascular histopathologic feature was frequently observed in MCD with TAFRO syndrome and it was decided to distinguish between MCD with regressed germinal center and hypervascularization without plasmacytosis as a hypervascular subtype (2, 37). However, although lymph node biopsy is strongly recommended, MCD-like histopathologic features were not necessarily included in the diagnostic criteria (38). Furthermore, overexpression of IL-6 and a cytokine storm is an important clinical finding of both MCD and TAFRO syndrome related to systemic symptoms, but other diseases such as hemophagocytic syndrome, IgG4-related disease, various infections or autoimmune diseases, or even after chimeric antigen receptor T-cell therapy also frequently occurs, making IL-6 elevation unsuitable for differential diagnosis (39). Therefore, the etiology and mechanism of TAFRO syndrome is currently unknown, and it has remained controversial whether TAFRO syndrome is a subset of MCD, distinct from MCD, or an overlapping entity with MCD (35–39). It would be helpful to further explore the pathophysiology of TAFRO syndrome, identify disease-specific biomarkers, and identify new therapeutic targets with a well-designed study in the future.

This study has some limitations. The retrospective nature and the possible selection bias from patients and data collection present the limitations of the current study. Furthermore, the heterogeneity in the MCD treatment modality in our cohort may limit the apparent therapeutic effect of siltuximab. Only 15 and 12 patients received siltuximab as first-line and salvage treatment, respectively, and analysis with such a small sample-sized cohort in a single-center setting might be less reliable. Consequently, further multi-center studies with larger sample sizes are warranted to generalize the findings to other populations. Our cohort also did not include HIV-positive patients with MCD, who may present with different clinical features and/or treatment outcomes. Moreover, we were unable to study serum IL-6 levels at diagnosis or during treatment, which are key cytokines in the pathophysiology of MCD. However, our cohort had a relatively large number of participants with a sufficient follow-up period to provide a real-world experience for various treatments for this rare disease condition in the Korean population.

There is still no consensus on when to stop the siltuximab treatment or prolong the infusion intervals, as the evidence for “end of treatment” of siltuximab is still scarce. When the patient achieves the best response after siltuximab treatment, the following approaches are considered: maintenance treatment with siltuximab for only a specific period; discontinuation of the siltuximab infusion; or continuous infusion of siltuximab with prolonged intervals of 6 to 8 weeks. However, in real-world practice, many patients develop MCD recurrence and progression, on the basis of their symptoms as well as laboratory and imaging findings, after stopping or prolonging siltuximab infusion. Therefore, a prospective study comparing the clinical, histopathologic, or immunologic differences between MCD patients with symptom resolution and those with symptom aggravation after cessation or prolongation of treatment will be valuable. Moreover, developing an alternative treatment for patients with siltuximab failure who usually show dismal prognosis and a standard treatment modality for HHV-8 positive MCD patients who were excluded from the pivotal trials is also crucial (27).

In conclusion, establishing a treatment strategy based on symptoms as well as laboratory, pathologic, and imaging findings of CD patients is crucial. Surgical resection is the primary treatment modality of UCD with solitary mass, and showed a better prognosis than with MCD, in which old age and splenomegaly are independent prognostic factors. The anti-IL-6 monoclonal antibody siltuximab is a novel therapeutic modality that is the preferred first-line therapy for MCD, especially iMCD, and it could improve the quality of life and prevent severe disease progression among MCD patients. Given the unmet need for treatment guidelines, well-designed prospective studies investigating the outcomes of the discontinuation or extension of siltuximab infusion interval, MCD molecular pathophysiology, and alternative therapeutic modality for the MCD patients who were refractory to siltuximab are required.

## Data availability statement

The data analyzed in this study is subject to the following licenses/restrictions: Privacy or ethical restrictions. Requests to access these datasets should be directed to S-GC, chosg@catholic.ac.kr.

## Ethics statement

The studies involving human participants were reviewed and approved by Institutional Review Board (IRB) of Seoul St. Mary’s Hospital. Written informed consent for participation was not required for this study in accordance with the national legislation and the institutional requirements.

## Author contributions

G-JM performed the research, collected and analyzed the data, and wrote the manuscript. Y-WJ, TK, DK, and JYL provided the patients and materials and reviewed the manuscript. S-SP, SP, and J-HY provided materials and reviewed the manuscript. S-EL, B-SC, Y-JK, H-JK, SL, C-KM, and JWL reviewed the manuscript and analyzed the data. K-SE and S-GC designed and conducted the study, provided patients and materials, analyzed data, and wrote the manuscript. All authors have read and approved the final manuscript.

## Acknowledgments

We wish to thank the members of the CULG (Catholic University Lymphoma Group) network for their valuable contribution to gathering patient information as well as the care of these patients in Multidisciplinary care service.

## Conflict of interest

The authors declare that the research was conducted in the absence of any commercial or financial relationships that could be construed as a potential conflict of interest.

## Publisher’s note

All claims expressed in this article are solely those of the authors and do not necessarily represent those of their affiliated organizations, or those of the publisher, the editors and the reviewers. Any product that may be evaluated in this article, or claim that may be made by its manufacturer, is not guaranteed or endorsed by the publisher.
