# Supplementary material for: Long-term treatment outcome of Castleman’s disease: A real-world experience
Source: Front Oncol. 2022 Aug 5;12:974770. doi: 10.3389/fonc.2022.974770 (PMC9389339; doi:10.3389/fonc.2022.974770)
Supplement: Supplementary file 1 [file DataSheet_1.docx]

Supplementary Materials

**Supplementary Table 1**. Univariate analysis of 54 patients with multicentric Castleman disease

| **Variables** † | **OS (95% CI)** | ***p-value*** | **PFS (95% CI)** | ***p*-value** |
| --- | --- | --- | --- | --- |
| **Age (years)** |  | *0.036* |  | *0.002* |
| <60 (n=43) | 92.6% (78.6–97.6) |  | 51.3% (24.1–73.1) |  |
| ≥60 (n=11) | 40.9% (1.32–83.2) |  | 27.3% (1.69–66.1) |  |
| **Sex** |  | 0.243 |  | 0.312 |
| Male (n=31) | 93.1% (74.9–98.2) |  | 53.1% (9.88–84.0) |  |
| Female (n=23) | 78.1% (49.0–91.8) |  | 37.2% (12.5–62.1) |  |
| **Histological subtype** |  | 0.557 |  | 0.159 |
| Hyaline vascular type (n=15) | 93.3% (61.3–99.0) |  | 0% |  |
| Plasma cell or Mixed type (n=39) | 83.3% (62.1–93.3) |  | 44.3% (21.5–65.0) |  |
| **ECOG** |  | *0.033* |  | 0.065 |
| 0-1 (n=44) | 92.8% (79.1–97.6) |  | 49.4% (18.8–74.3) |  |
| 2 (n=10) | 60.0% (16.4–86.5) |  | 30.% (4.93–61.8) |  |
| **Weight loss** |  | 0.082 |  | 0.106 |
| No (n=43) | 89.0% (65.5–96.8) |  | 53.7% (24.2–76.1) |  |
| Yes (n=11) | 71.6% (35.0–89.9) |  | 20.8% (1.06–58.1) |  |
| **Splenomegaly** |  | *0.012* |  | *0.016* |
| No (n=35) | 97.1% (81.4–99.6) |  | 59.8% (18.3–85.6) |  |
| Yes (n=19) | 68.2% (37.5–86.1) |  | 29.3% (8.23–54.7) |  |
| **Any effusions*** |  | 0.833 |  | 0.066 |
| No (n=38) | 89.4% (74.2–95.9) |  | 58.9% (30.1–79.2) |  |
| Yes (n=16) | 73.8% (24.5–93.7) |  | 0% |  |
| **Pulmonary involvement (GGOs)** |  | 0.558 |  | 0.063 |
| No (n=49) | 86.6% (68.3–94.8) |  | 53.5% (27.0–74.2) |  |
| Yes (n=5) | 80.0% (20.4–96.9) |  | 0% |  |
| **Hemoglobin (g/dL)** |  | 0.567 |  | *0.049* |
| Normal (n=16) | 93.3% (61.3–99.0) |  | 93.8% (63.2–99.1) |  |
| Anemia (n=38) | 84.0% (64.1–93.4) |  | 37.7% (16.0–59.5) |  |
| **CRP (mg/dL)** |  | 0.463 |  | 0.367 |
| Normal (n=22) | 86.1% (62.9–95.3) |  | 81.9% (58.5–92.8) |  |
| Elevated (n=32) | 87.5% (64.4–96.0) |  | 39.1% (16.1–61.6) |  |
| **ESR (mm/h)** |  | 0.406 |  | 0.082 |
| Normal (n=20) | 95.0% (69.5–99.3) |  | 90.0% (65.6–97.4) |  |
| Elevated (n=34) | 82.8% (62.3–92.7) |  | 37.5% (15.8–59.4) |  |
| **Albumin (g/dL)** |  | 0.506 |  | 0.099 |
| ≥3.5 (n=34) | 85.2% (55.7–95.7) |  | 50.6% (9.66–82.0) |  |
| <3.5 (n=20) | 84.7% (59.7–94.8) |  | 37.1% (12.9–61.9) |  |
| **Globulin (g/dL)** |  | 0.363 |  | 0.879 |
| ≥3.5 (n=27) | 92.6% (73.5–98.1) |  | 49.4% (21.0–72.7) |  |
| <3.5 (n=27) | 77.0% (44.0–92.1) |  | 32.7% (1.77–73.3) |  |
| **LDH (IU/L)** |  | 0.641 |  | *0.025* |
| Normal (n=40) | 89.5% (74.4–96.0) |  | 56.5% (27.9–77.4) |  |
| Elevated (n=14) | 74.3% (24.5–93.9) |  | 0% |  |
| **eGFR** |  | 0.056 |  | 0.362 |
| Normal (n=48) | 88.5% (69.3–96.0) |  | 44.2% (18.7–67.2) |  |
| Decreased (n=6) | 62.5% (14.2–89.3) |  | 41.7% (5.61–76.7) |  |

CRP, C-reactive protein; ECOG, European Cooperative Oncology Group; eGFR, estimated glomerular filtration rate; ESR, erythrocyte sedimentation rate; LDH, lactate dehydrogenase; OS, overall survival; PFS, progression-free survival

† Univariate analysis variables were selected based on prior literature on currently known or potential factors affecting survival outcomes according to the researcher's prediction.

* Any effusion represents either pleural effusion or ascites.


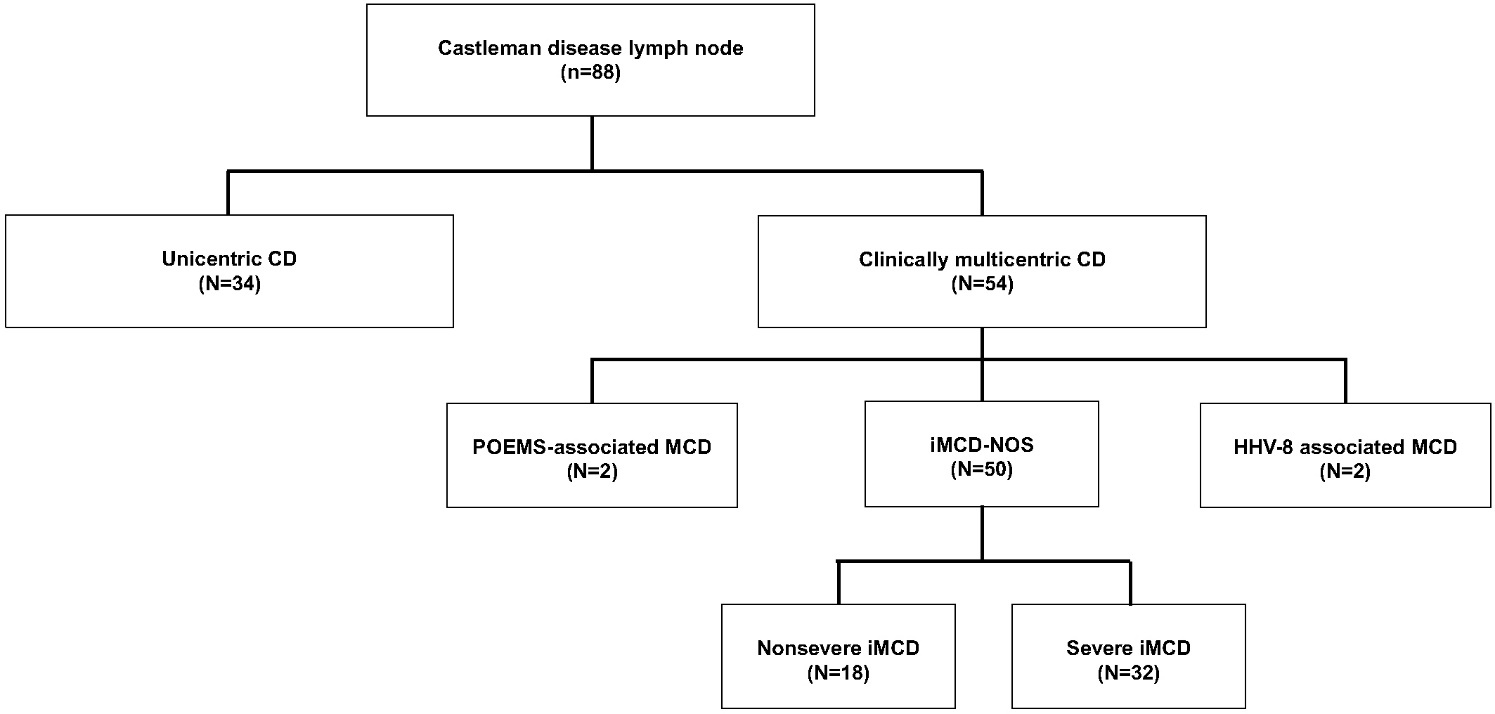


**Supplementary Figure 1**. _______________________________________________________

In this cohort, 34 (38.6%) and 54 (61.4%) patients had UCD and MCD, respectively. In the MCD group, 50 patients were identified as having idiopathic-MCD and 32 of them had severe idiopathic-MCD. Two patients were identified as having POEMS-associated MCD, and the other two were identified as HHV-8 positive. CD, Castleman disease; iMCD, idiopathic multicentric Castleman disease; iMCD-NOS, idiopathic multicentric Castleman disease – not otherwise specified; MCD, multicentric Castleman disease; POEMS, POEMS syndrome consists of polyneuropathy, organomegaly, endocrinopathy, monoclonal gammopathy, and skin changes; UCD, unicentric Castleman disease.
